# Management of Bone-Only Progressive Disease in Metastatic Breast Cancer—A Retrospective Single-Center Analysis

**DOI:** 10.3390/jcm15093456

**Published:** 2026-05-01

**Authors:** Christine Deutschmann, Paola Clauser, Florian Heinzl, Daphne Gschwantler-Kaulich, Christian F. Singer, Carmen Leser, Sabine Danzinger, Valentina Patrzek, Laura Anzengruber, Katharina Krepper, Georg Pfeiler

**Affiliations:** 1 Department of Obstetrics and Gynecology, Division of General Gynecology and Gynecologic Oncology, Medical University of Vienna, Waehringer Guertel 18-20, 1090 Vienna, Austria; 2 Department of Biomedical Imaging and Image-Guided Therapy, Medical University of Vienna, Waehringer Guertel 18-20, 1090 Vienna, Austria

**Keywords:** bone, oligoprogression, metastatic, breast cancer, bone-targeted agent, skeletal-related event

## Abstract

**Background/Objectives:** The optimal management of bone-only progressive disease (PD) in metastatic breast cancer remains unclear for several reasons. Radiologic diagnosis of bone PD is complicated by the lack of standardized response assessment criteria, unspecific morphologic changes of the bone, and flare-up phenomena. Furthermore, bone-only disease and oligoprogression have been associated with favorable prognosis challenging a change of systemic treatment with the consequence of limited treatment options in the future. Additionally, bone-only metastatic disease is frequently excluded from clinical trials resulting in scarce data. This study aimed to assess the therapeutic management and outcome of bone-only PD in metastatic breast cancer patients in a real-world academic setting. **Methods:** A retrospective analysis of all breast cancer patients with bone metastases (BMs) and at least one event of radiologic evidence of bone-only PD and/or the occurrence of a skeletal-related event (SRE) who were treated at the Department of Obstetrics and Gynecology of the Medical University of Vienna, Austria, between 1 January 2015 and 14 December 2021 was performed. In cases of multiple bone-only PD events in one patient only the first event was considered for analysis. All cases with PD in organs other than the bone were excluded. The primary outcome of the study was to assess therapeutic measures of bone-only PD. Secondary outcomes were the time from bone-only PD to next bone PD (TTF BD) and overall survival (OS; time from bone-only PD to death). Predictors of TTF BD and OS were assessed as exploratory outcomes. **Results:** Out of a total of 308 breast cancer patients with BMs, 57 had at least one event of bone-only PD. In 59.3% of bone-only PD cases the systemic treatment was continued with a numerically higher rate if multiple metastatic sites were present (71.4% vs. 46.4%). In most bone-only PD events the bone-targeted agent (BTA) was continued (94.5%), independent of the total number of metastatic sites. In 24.1% radiotherapy (RT) was administered with similar rates between patients with bone-only and multiple metastatic sites. The median TTF BD was 6.3 months. In multivariate analysis no predictor for TTF BD could be identified including change of systemic treatment, RT, previous BTA treatment duration, number of previous treatment lines for the metastatic disease, number of metastatic sites and previous or current SRE. Median OS was 21.8 months. Number of previous treatment lines for the metastatic setting was significantly associated with OS with shorter OS in the more advanced disease stage (*p*-value = 0.0208). **Conclusions:** Systemic and BTA treatment were continued in the majority of bone-only PD cases. In 24.1% RT was administered. No association between change of systemic therapy and improved oncologic outcome was found. The study’s results are hypothesis-generating in terms of whether change of systemic treatment should be performed restrictively to avoid limited treatment options in the future. Similarly, radiotherapy did not ameliorate prognosis.

## 1. Introduction

Approximately three-quarters of women with metastatic breast cancer develop bone metastases (BMs) [1,2].

BMs can cause skeletal-related events (SREs)—including pathological fractures, surgery and radiotherapy (RT) to the bone, spinal cord compression and hypercalcemia—with a one-year cumulative incidence of 38%. SREs potentially cause functional impairment, a decline in patient-reported quality of life and poor prognosis [3,4,5,6,7].

Treatment of BMs includes systemic treatment according to breast cancer subtype, anti-resorptive bone-targeted agents (BTAs), external beam radiation therapy (EBRT), targeted RT with systemic radioisotopes and orthopedic surgery [8].

BTAs are aimed at symptom palliation and prevention of SREs and disease progression. According to international guidelines BTAs should be started at the first diagnosis of BMs irrespective of whether they are symptomatic or not, be resumed in the event of disease progression and continued indefinitely into the hospice setting. BTAs vary in their mode of action. Bisphosphonates like zoledronic acid (ZA) inhibit osteoclastic-mediated activity by accumulation in the mineralized bone matrix and subsequent release during bone resorption. Furthermore, antitumor effects including inhibition of tumor cell migration, invasion, proliferation and viability have been discussed [9,10]. The BTA denosumab (Dmab) is a fully human monoclonal antibody that inhibits the receptor activator of NF-κB ligand (RANKL), inhibits osteoclastogenesis and decreases osteoclast-mediated bone resorption [11,12].

Local EBRT allows rapid pain relief of BMs with overall response rates (ORRs) of 70% to 80% [8]. A single 8 Gy fraction RT is recommended for painful uncomplicated BMs.

Targeted RT using systemic radioisotopes allows more precise delivery of the radiation doses to multiple tumor sites with relative sparing of normal tissues compared with EBRT. In breast cancer with osteoblastic skeletal metastases bone-seeking beta emitters such as samarium-153 lexidronam-labeled ethylenediaminetetramethylene phosphonic acid (^153^Sm-EDTMP) have shown efficacy in pain palliation.

In patients with good performance status and (impending) pathological fracture or (imminent) neural compression, orthopedic surgery is recommended in order to maintain patient functionality and mobility. Postoperative fractionated RT should be performed subsequently to prevent prothesis failure.

While disease progression in metastatic breast cancer generally prompts change of systemic treatment, the optimal therapeutic management of bone-only PD remains unclear for several reasons. Unspecific morphologic changes in the bone structure, flare-up phenomena and lack of standardized response assessment criteria complicate diagnosis of PD in the bone and leave the physician hesitant to change treatment but rather perform re-imaging follow-up [13,14,15,16]. Furthermore, non-measurable disease such as bone-only disease and oligoprogression (OP)—referring to tumor progression at 1–5 sites while other sites remain stable or respond to systemic therapy [17]—have been associated with favorable prognosis, challenging a change of systemic treatment with the consequence of limited treatment options for the patient in the future [16,18,19]. Furthermore, bone-only metastatic disease is frequently excluded from clinical trials due to the lack of measurable disease and the outstanding good prognosis—resulting in scarce data on the optimal therapeutic management of PD in the bone [16]. Additionally, while the BTA Dmab has demonstrated higher potency and a more advantageous drug profile than bisphosphonates, resulting in its preferential use in the first-line treatment of BMs and its continuation even in the event of disease progression, it has to be noted that its superior efficacy has only been demonstrated in Dmab-naïve patients [20]. The effect of a change of the BTA to a substance with a different mode of action in patients with progressive bone disease while on Dmab has yet to be determined.

This study aimed to assess the management of bone-only PD in metastatic breast cancer patients in a real-world academic setting. Furthermore, it evaluated time to next progressive bone disease (TTF BD), overall survival (OS) and determinants of the oncologic outcome.

## 2. Materials and Methods

A retrospective chart review was conducted to identify eligible patients, referring to all breast cancer patients with BMs with at least one event of bone-only PD—including radiologic evidence of bone-only PD and/or the occurrence of an SRE—who were treated at the Department of Obstetrics and Gynecology of the Medical University of Vienna, Austria, between 1 January 2015 and 14 December 2021. In cases of multiple bone-only PD events in one patient only the first event was considered for analysis. All cases with PD according to RECIST 1.1 in organs other than the bone were excluded.

Treatment response was assessed centrally in the course of routine clinical care every three to six months by clinical examination by board-certified gynecologists with an expertise in breast cancer treatment as well as by the institutional tumor board by review of radiologic images including computer tomography (CT), magnetic resonance imaging (MRI), positron emission tomography (PET CT), skeletal scintigraphy and ultrasound by board-certified radiologists specialized in breast cancer imaging.

A RECIST bone-only PD was defined as the appearance of bone lesions with a measurable soft tissue mass larger than 1 cm or an increase of 1 or more bone soft tissue metastases of more than 20% compared to the previous examination. A non-RECIST bone-only PD was defined as progressive osteoblastic or osteolytic BMs without a soft tissue component identified on CT, PET-CT and/or MRI, or a PD identified with bone-scan scintigraphy [21,22].

A SRE was defined as pathological fracture, surgery or RT to the bone, spinal cord compression or hypercalcemia [3].

Patient and disease characteristics were retrieved from patient charts.

The primary outcome of the study was to assess therapeutic measures of bone-only PD. Secondary outcomes were the TTF BD—defined as time between bone-only PD and next bone PD—and OS—defined as time from bone-only PD to death. The exploratory outcome was to evaluate predictors of the oncologic outcome including change of systemic treatment, RT, previous BTA treatment duration, number of previous treatment lines for the metastatic setting, number of metastatic sites and previous or current SREs.

Numerical variables are presented as median and interquartile range as well as mean and standard deviation (SD), while categorical data is given by absolute and relative frequencies. Time-to-event analyses were conducted using the Cox proportional hazards model with frailty terms to account for clustering at the patient level. Resulting estimates are given as odds ratios along with the respective 95% confidence interval. Change of treatment due to adverse events or progressive disease in another location than the bone, loss to follow-up, and death were handled as censoring events at the time of occurrence. These events were not considered to preclude the occurrence of the primary endpoint and were therefore treated as non-informative censoring within the modeling framework. A *p*-value below 0.05 was considered statistically significant. Statistical analysis was performed using R version 4.5.1.

## 3. Results

### 3.1. Demographics

**Figure 1:**
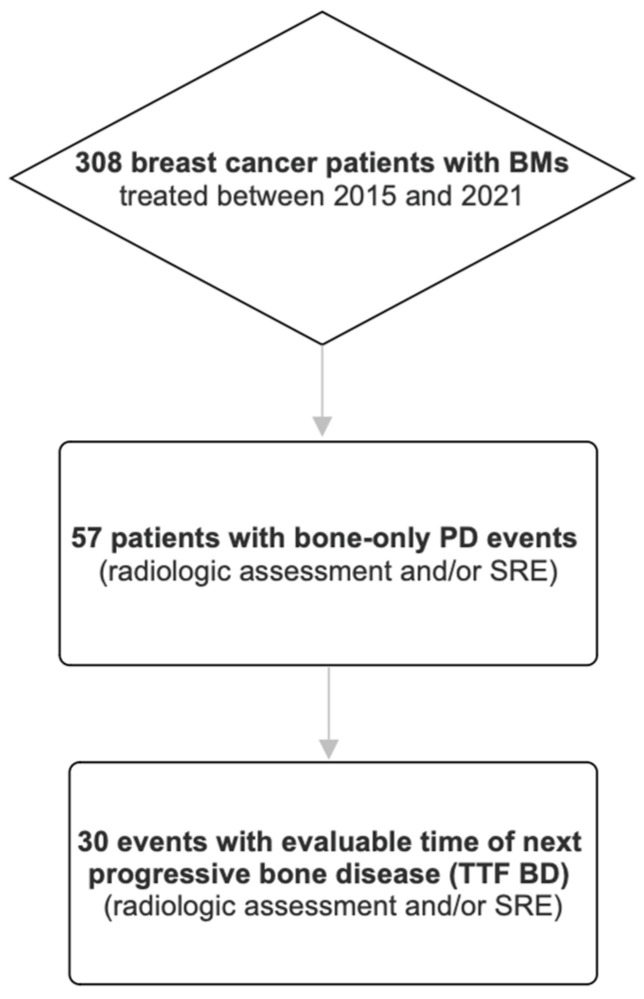
Study population overview. BMs: bone metastases, PD: progressive disease, SRE: skeletal-related event, TTF BD: time to next bone progressive disease.

We identified a total of 308 breast cancer patients with BMs who were treated at the Department of Obstetrics and Gynecology of the Medical University of Vienna, Austria, between 1 January 2015 and 14 December 2021 (see Figure 1). At a median follow-up duration (time between diagnosis of BMs and last clinical follow-up) of 44.6 months (1st quartile: 26.6; 3rd quartile: 77.6) per patient, 57 patients had at least one event of bone-only PD.

Most cases were estrogen receptor (ER)/progesterone receptor (PR)-positive, Human Epidermal Growth Factor Receptor 2 (HER2)-negative (78.6%) invasive ductal carcinomas (IDCs) (66.7%). The majority of patients had bone-only metastatic disease (59.6%). Patients received on average the first treatment line for the metastatic setting at the time of bone-only PD (1st quartile: 1; 3rd quartile: 2). The majority of patients (96.5%) received Dmab as BTA at the time of bone-only PD with a median previous treatment duration of 8.4 months (1st quartile: 4.3; 3rd quartile: 20.2); 57.1% had a previous SRE of any type. Further patient, disease and treatment characteristics are shown in Table 1 and Table 2.

### 3.2. Bone-Only Progressive Disease Diagnosis

In the cohort, 54.4% (31/57) of bone-only PD events were diagnosed by imaging only, 38.6% (22/57) by imaging and presence of an SRE, and 7.0% (4/57) by occurrence of an SRE only (see Table 3). Approximately 58.9% of bone-only PD events were diagnosed by CT scan. The majority of radiologic bone-only PD diagnoses did not fulfill RECIST criteria (62.3%), compared to 26.4% meeting RECIST criteria. Meanwhile, 11.3% fulfilled RECIST as well as non-RECIST criteria. Pain was the most common SRE (35.1%) followed by fracture (15.8%) and RT (15.8%) and spinal cord compression (1.8%) and surgery (1.8%).

### 3.3. Management of Bone-Only Progressive Disease

In 59.3% of bone-only PD cases the systemic treatment was continued with a numerically higher rate if multiple metastatic sites were present (71.4% vs. 46.4%, *p* = 0.080). Notably, systemic treatment line showed no significant association with change of systemic treatment at the time of bone-only PD (1st line: 38.9% (14/36) vs. ≥2nd line: 44.4% (8/18), *p* = 0.695).

In most bone-only PD events the BTA was continued (94.5%), independent of the total number of metastatic sites (bone-only mBC: 96.6% vs. multiple metastatic sites: 90.5%).

In 24.1% RT was administered in the event of bone-only PD, with similar rates between patients with bone-only and multiple metastatic sites (17.2% vs. 25.0%).

Table 4 summarizes the management measures for bone-only PD.

### 3.4. Oncologic Outcome and Predictors of Oncologic Outcome

The median duration between bone-only PD and last follow-up was 25.8 months (1st quartile: 12.2; 3rd quartile: 57.5). In 30 cases the time of next progressive bone disease could be evaluated (see Figure 1); of these 33.3% were diagnosed by radiologic assessment and presence of an SRE, 33.3% by occurrence of an SRE only and 33.3% by radiologic assessment only (see Table 5). In 4 cases no bone PD occurred until the last clinical follow-up and in 23 cases next bone PD could not be evaluated for other reasons. The median TTF BD was 6.3 months (1st quartile: 4.1; 3rd quartile: 16.0). In multivariate Cox regression analysis none of the following parameters were associated with TTF BD: change of systemic treatment (*p*-value = 0.2072), RT (*p*-value = 0.3331), previous BTA treatment duration (*p*-value = 0.3005), number of previous treatment lines for the metastatic setting (*p*-value = 0.1648), number of metastatic sites (*p*-value = 0.2326) and previous or current SREs (*p* = 0.0992) (see Figure 1).

**Figure 2:**
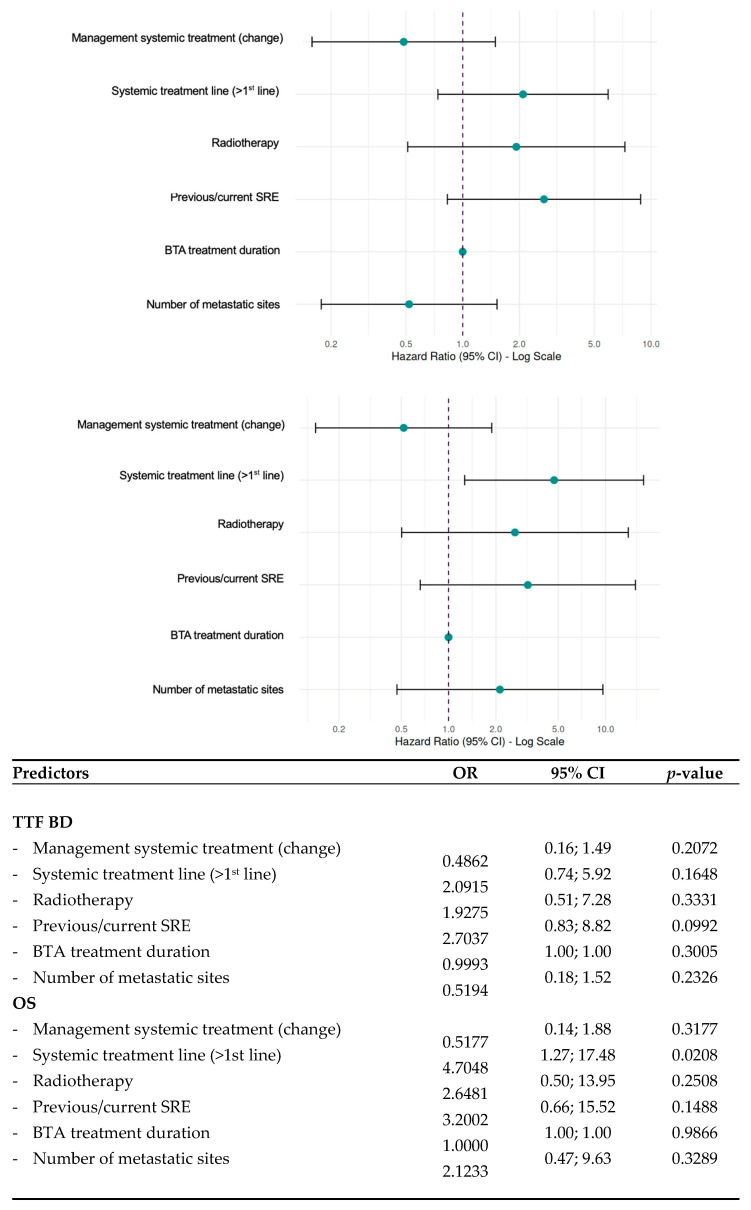
Predictors of time to next bone progressive disease (TTF BD) and overall survival (OS) in multivariate Cox regression analysis (upper image: TTF BD, lower image: OS). SRE: skeletal-related event, BTA: bone-targeted agent, OR: odds ratio, CI: confidence interval, TTF BD: time to next bone progressive disease, OS: overall survival.

Out of 57 patients, 28 had died at the time of last follow-up. Median OS was 18 months (1st quartile: 9.5; 3rd quartile 50.5). The number of previous treatment lines for the metastatic setting was significantly associated with OS with shorter OS in the more advanced disease stage (mean OS_1st treatment line_ = 33.1 months versus mean OS _≥ 2nd treatment line_ = 31.7 months) (*p*-value = 0.0208) (see Figure 2). None of the following other parameters were predictive of OS: change of systemic therapy (*p*-value = 0.3177), RT (*p* = 0.2508), previous BTA treatment duration (*p*-value = 0.9866), number of metastatic sites (*p*-value = 0.3289) and previous or current SREs (*p*-value = 0.1488).

## 4. Discussion

As new anticancer treatments extend OS of breast cancer patients, long-term management of metastatic bone disease is increasingly relevant [23]. OP is more and more observed owing to the extensive use of targeted treatments that potentiate the evolution of drug-resistant subclones [17,24]. While PD in metastatic breast cancer generally prompts change of treatment, the optimal management of bone-only PD remains unclear owing to challenging diagnostic conditions, the per se favorable prognosis of bone-only disease and OP as well as the limited number of clinical trials in this setting. The present study aimed to assess the management and outcome of bone-only PD in a real-world academic context.

In total 57 events of bone-only PD in 308 breast cancer patients with BMs were identified. The majority of bone-only PD events in the present study were diagnosed by imaging (93.0%). The most frequent radiologic diagnostic method used for bone evaluation was CT (58.9%), followed by MRI (19.6%), PET CT (16.1%) and skeletal scintigraphy (3.6%). Breast cancer guidelines comprise various imaging technologies with differing performance to detect and monitor BMs—including structural modalities such as plain radiographs, CT, MRI and metabolic and molecular imaging tools such as diffusion-weighted MRI (DW-MR), PET and single-photon emission CT (SPECT) [8,25,26,27,28]. Notably, evaluation of therapeutic responses of BMs is based on signs of bone repair and destruction rather than on changes in tumor volume, impairing objective quantification [8]. Furthermore, unspecific structural changes in the bone and flare-up phenomena complicate diagnosis of PD. In this context, bone-only PD in the present study was diagnosed based on heterogenous criteria. The majority of radiologic bone-only PD diagnoses did not fulfill RECIST criteria (62.3%), compared to 26.4% that fulfilled RECIST criteria. Meanwhile, 11.3% fulfilled RECIST as well as non-RECIST criteria. A high discordance of radiologic response assessment between investigators and blinded independent central review in bone-only metastatic disease (39.9%) and bone and other metastatic locations (47.5%) was described previously in a pooled analysis by the Food And Drug Administration [16]. To account for the challenging diagnostic conditions of BMs, the Prostate Cancer Working Group has recommended to confirm bone progressive disease with the evidence of new lesions in two sequential scans [29].

In 45.6% of bone-only PD events in the present study an SRE was present. Notably, SRE risk increases with the occurrence of an SRE as well as progression of the overall and bone metastatic disease and therefore aids in the diagnosis of bone PD [3,30,31,32].

Regarding the therapeutic management of bone-only PD, systemic treatment was continued in 59.3% of bone-only PD cases with a numerically higher rate if multiple metastatic sites including lymph node and visceral metastases that responded to treatment were present (71.4% vs. 46.4%, *p*-value = 0.080). Notably, a change of systemic treatment in the present study did not improve prognosis. This is hypothesis-generating in terms of whether change of systemic therapy should be performed restrictively in the event of bone-only PD to prevent limited treatment options in the future. The majority of patients (96.5%) received Dmab as BTA which was continued at the time of bone-only PD in most of the cases (98.1%). This is not surprising as Dmab has been shown to be superior to bisphosphonates such as ZA in preventing SREs [20,33,34,35,36,37,38,39,40,41,42,43,44,45,46]. It is characterized by a more rapid onset and longer duration of action than bisphosphonates. The significantly reduced risk of SREs with Dmab compared to ZA was shown to be independent of various patient and disease characteristics [33,47]. Furthermore, Dmab is associated with greater treatment adherence compared to bisphosphonates potentially owing to the subcutaneous route of administration and a more favorable tolerability profile [48]. Moreover, Dmab has demonstrated a beneficial cost-effectiveness ratio. The higher potency and more advantageous drug profile of Dmab compared to bisphosphonates has led to its common preferential use in the first-line treatment of BMs and its continuation even in the event of disease progression. Yet, it has to be noted that the superior efficacy of Dmab has only been demonstrated in Dmab-naïve patients. Especially in the event of OP—where the primary therapeutic aim is to eradicate treatment-resistant clones to prolong disease control—change of the BTA to a substance with a different mode of action and continuation of an otherwise effective systemic agent could be advantageous [49], particularly as change of the systemic treatment following bone-only PD showed no association with TTF BD and OS in multivariate Cox regression analysis in the present study and is therefore likely not a preferable therapeutic management option. Few studies have evaluated a change of the BTA in case of progressive bone disease [35,43,50]. Lipton A et al. reported, for instance, positive outcomes following a change from ongoing intravenous bisphosphonate therapy (ZA or pamidronate) to Dmab in the presence of elevated urinary N-telopeptide (uNTx) levels (representing excessive bone resorption and functioning as a surrogate marker for SREs, cancer progression and death) [51], whereas continuation of the bisphosphonate treatment only led to progression and lower decrease in uNTX levels. Owing to the small sample size the effect of a change of the BTA in the event of bone-only PD could not be evaluated in the present study.

In 24.1% RT was administered at the time of bone-only PD with similar rates between patients with bone-only and multiple metastatic sites. Few studies with divergent results exist on stereotactic RT (SBRT). The CURB trial showed no difference in progression-free survival (PFS) with the addition of SBRT to standard palliative systemic treatment in breast cancer patients with oligoprogressive disease [52]. The AVATAR trial demonstrated that SBRT to oligoprogressive lesions in ER-positive/HER2-negative breast cancer while on treatment with CDK4/6 inhibitors allowed continuation of systemic therapy in a clinically meaningful number of patients [53]. The discrepant results of the two trials can be explained by their differences in patient and disease characteristics. While the AVATAR trial only included hormone receptor-positive/HER2-negative breast cancer, the CURB trial studied all subtypes, in particular 34% TN breast cancer cases. Furthermore, the CURB trial included patients in a later disease setting with 3–4 previous lines of therapy compared to only 1–2 lines in the AVATAR trial [49]. In the present study RT was not associated with improved TTF BD and OS. Yet, as details on the indication of RT (pain control, impending complications or oligoprogression control), dose, fractionation and technique (conventional palliative RT or SBRT) could not be defined in the present study owing to the study’s retrospective nature, interpretation of the oncologic impact of RT is limited.

The present study did not identify predictors for TTF BD and OS including therapeutic measures such as change of systemic treatment and RT or disease and treatment characteristics such as previous BTA treatment duration, number of metastatic sites and previous or current SREs. Only the number of previous treatment lines for the metastatic setting was significantly associated with OS with shorter OS in the more advanced disease stage (*p*-value = 0.0208). In comparison, previous studies identified multiple BMs, metastases both in the axial and appendicular skeleton, concurrent SRE, age under 40 or above 49, and black/non-Hispanic ethnicity as predictors for poorer OS [54,55,56]. In conclusion, additional prognostic and predictive biomarkers as well as novel treatment options are needed to optimize the management of bone-only PD.

Limitations of the study include differing re-imaging intervals in each patient, heterogenous diagnostic approaches and use of response criteria. This limits the reproducibility and external validity of the study results. As a result of the small sample size and retrospective nature of the study only a limited set of clinically relevant variables could be considered in the Cox proportional hazards model. Therefore, residual confounding cannot be excluded. Furthermore, the lack of a more detailed discussion of RT including indication, dose, fractionation and technique limits the informative value of the impact of RT on the oncologic outcome.

## 5. Conclusions

In conclusion, the present study evaluated the management of bone-only PD in a real-world academic setting. Bone-only PD was diagnosed based on heterogenous parameters including RECIST, non-RECIST criteria and symptom burden. In the majority of bone-only PD events systemic treatment and BTA were continued. Change of systemic treatment did not improve oncologic outcome. This is hypothesis-generating in terms of whether change of systemic therapy should be performed restrictively to avoid limited treatment options in the future. Similarly, radiotherapy did not ameliorate prognosis. Additional treatment options and prognostic and predictive biomarkers are needed to optimize bone-only PD outcome.

## Figures and Tables

**Table 1:** Patient and disease characteristics.

Demographics	Number of Patients with at Least One Event of Bone-Only PD n = 57
**Age**, median (1st quartile; 3rd quartile)	60 (51; 67)
**Timing of metastatic disease**	
- De novo	24 (42.1%)
- Secondarily metastasized	33 (57.9%)
**Time between diagnosis of primary tumor and metastatic bone disease**,	
months, median (1st quartile; 3rd quartile)	15.4 (0.7; 80.0)
Histology *	
- IDC	38 (66.7%)
- ILC	17 (29.8%)
- Invasive mucinous carcinoma	1 (1.8%)
- Invasive carcinoma no further classification	1 (1.8%)
Subtype *	
- ER/PR +, HER2-	44 (78.6%)
- ER/PR +, HER2+	3 (5.4%)
- ER/PR −, HER2+	2 (3.6%)
- TN	3 (5.4%)
- ER/PR +, HER2 unknown	4 (7.1%)
- Missing data	1

* based on biopsy of the primary tumor in the breast, PD: progressive disease, IDC: invasive ductal carcinoma, ILC: invasive lobular carcinoma, ER: estrogen receptor, PR: progesterone receptor, HER2: Human Epidermal Growth Factor Receptor 2, TN: triple negative.

**Table 2:** Disease and treatment characteristics at the time of bone-only PD.

Disease and Treatment Characteristics (at Time of Bone-Only PD)	Number of Bone-Only PD Cases n = 57
**Tumor locations**	
- Bone only	31 (59.6%)
- Oligometastatic bone disease (1–5 BMs)	16
- Multiple bone metastases (>5 BMs)	15
- Multiple metastatic sites	21 (40.4%)
- Missing data	5
**Type of bone metastases**	
- Osteoblastic	33 (57.9%)
- Osteolytic	15 (26.3%)
- Mixed	9 (15.8%)
**Previous SRE any type**	
Missing data n = 1	32 (57.1%)
**Ongoing BTA at the time of bone-only PD**	
- Denosumab	55 (96.5%)
- Zoledronic acid	1 (1.8%)
- Other bisphosphonate	1 (1.8%)
**Previous treatment duration of BTA**, months, median (1st; 3rd quartile)	8.4 (4.3; 20.2)
**Treatment line at the time of bone-only PD** (median (1st; 3rd quartile)	
Missing data n = 1	1 (1; 2)

PD: progressive disease, BMs: bone metastases, SRE: skeletal related event, BTA: bone-targeted agent.

**Table 3:** Bone-only progressive disease diagnosis.

Bone-Only Progressive Disease Diagnosis	Total Study Population n = 57	According to Imaging and SRE n = 22	According to Imaging Only n = 31	According to SRE Only n = 4
**Imaging diagnostic criteria** - RECIST criteria fulfilled - Non-RECIST criteria fulfilled - RECIST and Non-RECIST criteria fulfilled	14 (26.4%) 33 (62.3%) 6 (11.3%)	9 (40.9%) 10 (45.5%) 3 (13.6%)	5 (16.1%) 23 (74.2%) 3 (9.7%)	NA NA NA
**Imaging method to diagnose bone-only PD** - CT - MRI - PET CT - Skeletal scintigraphy - Ultrasound - Missing data	33 (58.9%) 11 (19.6%) 9 (16.1%) 2 (3.6%) 1 (1.8%) 1	13 (59.1%) 5 (22.7%) 2 (9.1%) 1 (4.5%) 1 (4.5%) 0	19 (63.3%) 4 (13.3%) 6 (20.0%) 1 (3.3%) 0 1	NA NA NA NA NA NA
Type of SRE * - Pain - Fracture - Radiotherapy - Spinal cord compression - Surgery	20 (35.1%) 9 (15.8%) 9 (15.8%) 1 (1.8%) 1 (1.8%)	16 (72.7%) 9 (40.9%) 7 (31.8%) 1 (4.5%) 1 (4.5%)	NA NA NA NA NA	4 (100%) 0 2 (50%) 0 0

* multiple answers possible, SRE: skeletal-related event, RECIST: Response Evaluation Criteria in Solid Tumors, NA: not applicable, PD: progressive disease, CT: computer tomography, MRI: magnetic resonance imaging, PET CT: positron emission tomography.

**Table 4:** Management of bone-only progressive disease.

Management Measures	Number of Bone-Only PD Cases n = 57		
	Total Study Population n = 57	Bone-Only mBC n = 31	Multiple Metastatic Sites n = 21
**BTA**			
- **Change**	3 (5.5%)	1 (3.4%)	2 (9.5%)
- Zoledronic acid to denosumab	1	0	1
- Other bisphosphonate to denosumab	1	1	0
- Denosumab to zoledronic acid	1	0	1
- **Continuation**	52 (94.5%)	28 (96.6%)	19 (90.5%)
- **Not evaluable**	2	2	0
- No information	1	1	0
- Patient died prior to therapy	1	1	0
**Systemic treatment**			
- **Change**	22 (40.7%)	15 (53.6%)	6 (28.6%)
- **Continuation** - **Not evaluable** - No information - Patient died prior to therapy	32 (59.3%) 3 2 1	13 (46.4%) 3 2 1	15 (71.4%) 0 0 0
**Other treatments**			
- **Radiotherapy**	13 (24.1%)	5 (17.2%)	5 (25%)
- **Samarium**	1 (1.9%)	NA	NA
- **None**	40 (74.1%)	24 (82.8%)	15 (75%)
- **Not evaluable**	3	2	1
- No information	1	1	0
- Patient died prior to therapy	2	1	1

PD: progressive disease, mBC: metastatic breast cancer, BTA: bone-targeted agent, NA: not applicable.

**Table 5:** Oncologic outcome.

Oncologic Outcome	Number of Bone-Only PD Cases n = 57
**Next bone PD**	
- **Radiologic assessment only**	10 (33.3%)
- SRE only *	10 (33.3%)
- Pain	9
- Radiotherapy	5
- Radiologic assessment and SRE *	10 (33.3%)
- Pain	8
- Radiotherapy	4
- Fracture	2
- Spinal cord compression	1
- **Not evaluable**	27
- Change of treatment	16
- Due to AE	8
- Due to PD in other location than bone	8
- Loss to follow-up	2
- Patient died due to unknown cause	5
- No PD until last follow-up date	4

* multiple answers possible, PD: progressive disease, SRE: skeletal-related event, AE: adverse event, PD: progressive disease.

## Data Availability

Data is unavailable for sharing due to privacy restrictions.

## Abbreviations

The following abbreviations are used in this manuscript:

AE	Adverse event
BM	Bone metastasis
BTA	Bone-targeted agent
CI	Confidence interval
CT	Computer tomography
Dmab	Denosumab
EBRT	External beam radiation therapy
ER	Estrogen receptor
FUP	Follow-up
HER2	Human Epidermal Growth Factor Receptor 2
IDC	Invasive ductal carcinoma
ILC	Invasive lobular carcinoma
MRI	Magnetic resonance imaging
NA	Not applicable
OP	Oligoprogression
OR	Odds ratio
ORR	Objective response rate
OS	Overall survival
PD	Progressive disease
PFS	Progression-free survival
PR	Progesterone receptor
PET CT	Positron emission tomography
RANKL	Receptor activator of NF-κB ligand
RECIST	Response Evaluation Criteria In Solid Tumors
RT	Radiotherapy
SBRT	Stereotactic radiotherapy
SD	Standard deviation
SPECT	Single-photon emission CT
SRE	Skeletal-related event
TN	Triple negative
TTF BD	Time to next bone progressive disease
uNTX	Urinary N-telopeptide
ZA	Zoledronic acid
